# Regeneration or Scarring Derive from Specific Evolutionary Environmental Adaptations of the Life Cycles in Different Animals

**DOI:** 10.3390/biology12050733

**Published:** 2023-05-17

**Authors:** Lorenzo Alibardi

**Affiliations:** Comparative Histolab Padova and Department of Biology, University of Bologna, 40126 Bologna, Italy; lorenzo.alibardi@unibo.it

**Keywords:** animal regeneration, evolution, metamorphosis, genes, regenerative medicine

## Abstract

**Simple Summary:**

The present MS proposes a new hypothesis that motivates why only some animals can regenerate their organs. This derives from their simple body constitution, environment where they live, evolutionary story and life cycles. Only water-dwelling animals or animals that include drastic body changes in their life (metamorphosis) can regenerate when they are adults after an injury or organ-loss. This derives from the re-utilization of numerous genes that were previously utilized for metamorphosis. Humans and the remaining terrestrial animals have lost or altered the functionality of these genes during their evolution, and therefore cannot regenerate anymore. Whether regenerative gene therapies can help to improve human regeneration is a big challenge for the future of regenerative medicine.

**Abstract:**

The ability to heal or even regenerate large injuries in different animals derives from the evolution of their specific life cycles during geological times. The present, new hypothesis tries to explain the distribution of organ regeneration among animals. Only invertebrates and vertebrates that include larval and intense metamorphic transformations can broadly regenerate as adults. Basically, regeneration competent animals are aquatic while terrestrial species have largely or completely lost most of the regeneration ability. Although genomes of terrestrial species still contain numerous genes that in aquatic species allow a broad regeneration (“regenerative genes”), the evolution of terrestrial species has variably modified the genetic networks linking these genes to the others that evolved during land adaptation, resulting in the inhibition of regeneration. Loss of regeneration took place by the elimination of intermediate larval phases and metamorphic transformations in the life cycles of land invertebrates and vertebrates. Once the evolution along a specific lineage generated species that could no longer regenerate, this outcome could not change anymore. It is therefore likely that what we learn from regenerative species will explain their mechanisms of regeneration but cannot or only partly be applied to non-regenerative species. Attempts to introduce “regenerative genes” in non-regenerative species most likely would disorder the entire genetic networks of the latter, determining death, teratomas and cancer. This awareness indicates the difficulty to introduce regenerative genes and their activation pathways in species that evolved genetic networks suppressing organ regeneration. Organ regeneration in non-regenerating animals such as humans should move to bio-engineering interventions in addition to “localized regenerative gene therapies” in order to replace lost tissues or organs.

## 1. Animal Regeneration Derives from the Development of Specific Life Cycles

**Figure 1:**
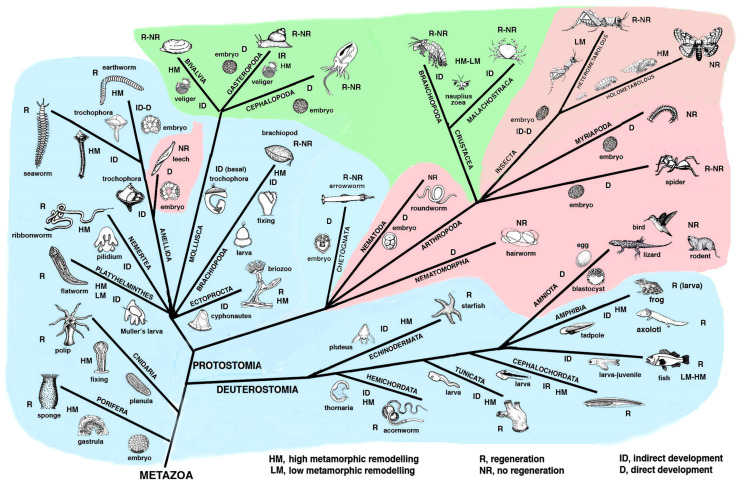
Schematic simplified tree reporting the different phases of the life cycle present in main metazoan groups (phyla or classes) associated with their regenerative ability. A large or more distributed regeneration occurs in aquatic metazoans (light blue background) with relatively simple life cycles or those that include larval forms and intense metamorphic transformations during their life. The green area comprises species with limited regeneration, mainly on their body appendages (legs, arms, shell, eye stalk etc.). The pink areas include species unable to regenerate, aside from a few cases (legs in growing spiders, the tail in lizards). In nematodes, numerous insects, some crustaceans, myriapods, some fish etc., the larval transitions mainly occur only through the somatic growth of their organs and of the entire body size with a small re-organization of their anatomy. The larval stages of numerous of these metazoans are simply miniaturized forms of their adult phase when they become sexually mature. In some holometabolous insects the last stages (pupa, chrysalis) are very different from previous larval stages and also from the adult stage. However, most changes derive from regional “stem cell sacs” called imaginal discs and not from a broad distribution of replacing cells. Finally, in numerous species of fishes, not only one but two or even more dramatic metamorphic events occur so that their bodies undergo complete morphological and physiological changes, and regeneration is very active as adults.

Once fully developed as an adult, an animal can incur small or large injuries, including the loss of body regions such as an arm, a leg, an eye, a kidney and so forth. The body senses the loss and repairs the damage through scarring in some animals while in others a regenerative process allows to anatomically and functionally restore the damaged organ. In the animal world regeneration abilities are very variable so that we can say that there are broad regenerators, medium–low regenerators and species that cannot regenerate [1,2,3,4,5,6,7] (Figure 1).

Why can some animals regenerate while others, in particular humans, cannot? This question should initially consider that a hydra or a planaria, the champions of regeneration, can broadly regenerate since they are simple multicellular organisms, although some species may have large genomes or genomes similar to those of more morphologically complex animals [8]. In fact, genome size and number of functional genes are not correlated for numerous species and large genomes contain numerous repetitive DNA sequences with few active genes, especially for development. Therefore, genome coding for developing simpler anatomical planes have ample regeneration capacity while more complex bodies derived from large functional genomes are present in animals that cannot regenerate. The developmental, anatomical and physiological complexity of a vertebrate is much higher from those of a hydra, a flatworm, a ribbonworm, a polychete anellid or a seasquirt and justify the poor regeneration of the former versus the high regeneration ability of the latter.

**Figure 2:**
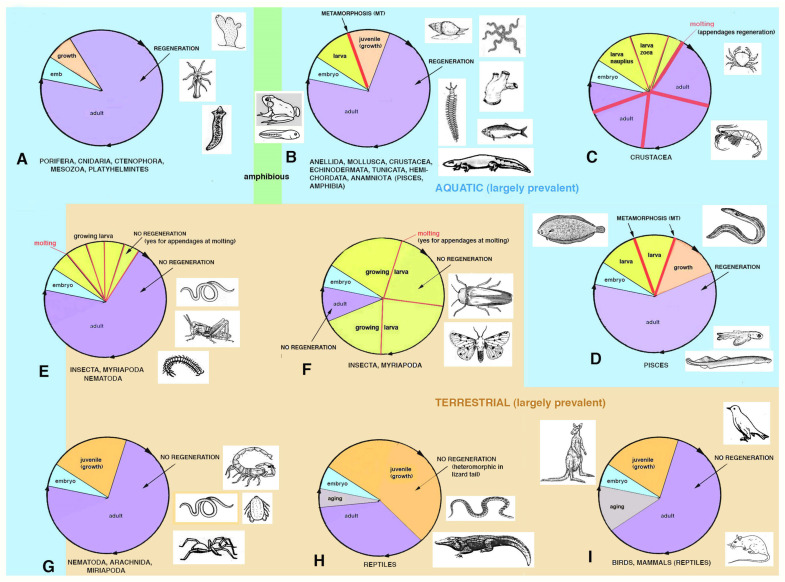
Schematic and generalized drawing indicating the different phases within the life cycles of different metazoa, roughly subdivided into aquatic (pale blue background) and terrestrial (pale brown background) and amphibious (green). Some cycles synthetically comprise the same groups of animals, but other specific details are not represented. In each cycle, different names and colors (wedges) represent successive phases (following the circular arrows from the embryo) of the cycle. The adult phase (sexually mature) is indicated in violet color and only in mammals and some reptiles is the aging phase also indicated (in grey color). (**A**) is the relatively simple cycle of lower animals with high regeneration. (**B**) represents the more complex cycles of numerous invertebrates and some anamniote vertebrates with a larva stage and metamorphosis and with high regeneration. (**C**) is the life cycle of crustaceans including larval stages and metamorphosis and some regeneration during and after molting (mainly in appendages). (**D**) is the life cycle of numerous fishes, with at least one but often more larval stages with growth and sometimes dramatic metamorphic events. They are also able to regenerate various organs. (**E**) represents the life cycle of some arthropods and nematodes with larval phases, small metamorphic changes and mainly growth after a number of molts. They do not regenerate or only regenerate body appendages. (**F**) is the modified cycle of some arthropods, mainly insects, with a short adult phase and a variable number of larval phases mainly concerning growth. Appendage regeneration occurs only after molting. (**G**) is the life cycle of other arthropods and nematodes that, after hatching, show continuous growth with little regeneration or evident molting phases and no regeneration. (**H**) is the cycle of ectothermic amniotes (reptiles) with no larval and metamorphic phases but continuous growth until most or a large part of the adult phase. They cannot regenerate (aside from the lizard tail) and some aging is evident in some species. (**I**) shows the life cycle of birds and mammals that includes a terminal, variably long and evident phase of aging and no regeneration.

Despite the above consideration, it is very important to learn the mechanisms of regeneration from regenerative species in the hope that this knowledge will allow translating these mechanisms into non-regenerative animals. This question has activated a vast amount of research in the last 150 years and, lately, on cellular, molecular biology and on the genetics of regeneration [2,3,9,10]. The final goal of these investigations is the discovery of molecular clues from regenerating animals and to apply this knowledge to improve human regeneration [11,12]. Although the basic molecular mechanisms will ultimately explain the ample regeneration of competent organisms, most physicians and biologists neglect to see general basic biological reasons for the presence of regeneration only in some animals and its absence in others [1,5]. The noted differences among species reflect how complex development occurs in different environments and the anatomical organization present in diverse animals as adults. The latter is an adaptation to the environment during the progressive phases of the biological cycle in each species—cycles that derive from a long evolutionary history (Figure 2).

Figure 1 and Figure 2 summarize the distribution of regeneration among metazoans, noting that most regenerative species are linked to the water environment and are mainly marine animals. Numerous low forms of marine animals such as sponges, flatworms, ribbonworms and ctenophores have less complex bodies in comparison to those of terrestrial animals, particularly those of arthropods and vertebrates. However, a relatively high regenerative ability is also present in more complex animals, such as sea slugs, sea cucumbers, starfish, seasquirt, numerous fishes, caudate amphibians and frog tadpoles—all animals linked to the sea, freshwater or humid environments and that have metamorphosis in their life cycle (Figure 2). Other animals such as earthworms, octopus, sea snails, numerous crustaceans and many fishes, also living in water or humid environments but missing drastic meta-morphic transitions, can repair or even regenerate few organs. Another group of terrestrial animals such as spiders, some crustaceans and insects with progressive growth through molts, can only regenerate their appendages but little or none of their inner organs (nervous ganglia, heart, intestine, hepatopancreas, gonads etc.). Finally, other terrestrial animals such as roundworms and scorpions, and all terrestrial vertebrates (amniotes) such as reptiles, birds and mammals, cannot regenerate at all, aside from healing their wounds by scarring and occasionally through “*regengrow*” [1,4,5,13,14,15,16,17,18]. The term *regengrow* indicates a healing or even regenerative process that occurs while the animal is still variably growing, and this concept has been broadly explained in previous studies [18,19]. Broad regeneration only occurs in very hydrated conditions and this process is much more common in sea- or freshwater-adapted animals than in terrestrial adapted animals (Figure 1 and Figure 2). This depends on the formation of a regenerative blastema, a soft and hydrated environment for cell multiplication, movements and for the special re-organization that would be hampered in denser and drier tissues typically found in post-embryonic terrestrial animals.

The present analysis is hypothetical and is based on general biological considerations derived from the specific evolutionary trajectories taken by different phyla (Figure 1), but the molecular developmental details of the specific evolution in each group of animals represent a huge effort for next generations of biologists.

## 2. Distribution of Regeneration among Animals

Lower invertebrates include acelomates (Poriphera, Cnidaria, Ctenophora, Platyhelminthes, Nemerteans), pseudocelomates (Nematods, Nematomorphs, Rotifers, Achanthocephals, Gastrotrics, Ectoprocts, and other minor groups) and the remaining celomates (mainly Anellids, Molluscs, Arthropoda, Echinoderms and Chordates; Figure 1). Pseudocelomates and celomates have reached an organ level organization based on a general increase of their genomes and gene regulation [8]. The biological cycles are variable, including those of anellids, molluscs, arthrophods, echinoderms and chordates (Figure 2). During evolution, most of these phyla of animals initiated their biological cycles with a primitive, indirect development with a trochophora or other natant types of larvae, but some evolutionary lineages later shortened or suppressed these larval stages that were replaced by a direct development for the terrestrial adaptation [20].

**Figure 3:**
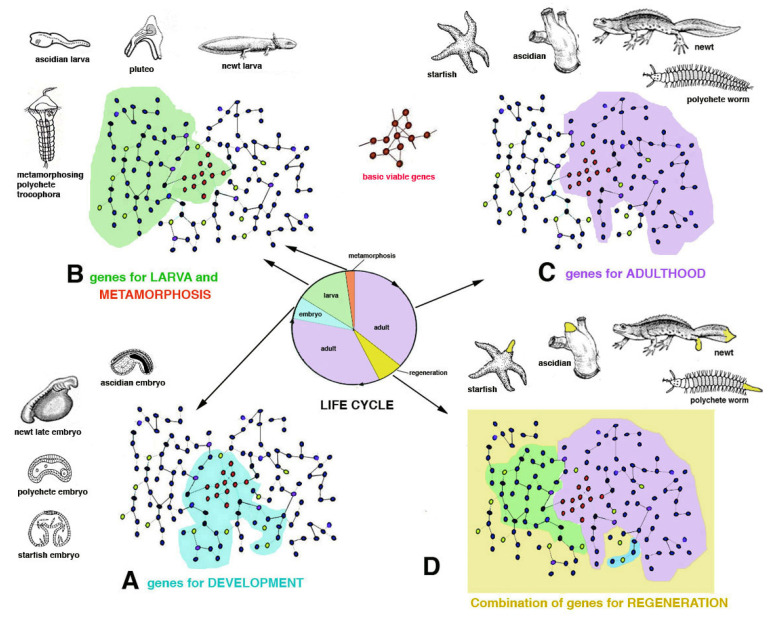
Hypothetic scheme referring to the usage of “different areas” (different gene pathways) of the genome in four different animals that present larvae and metamorphosis in their life cycle (the colored circle) and regeneration in adults. The central region of the hypothetic genomes (in red circles) contains the “basic viable genes” that are used throughout the life cycle. Due to space limitation, the indicative genomes are represented in the same way for the four animals although they would be very different in reality. During development (**A**), some genes are used (light blue-colored area within the life cycle). In the larval stage and metamorphosis (**B**), other and some common genes are also utilized (green-colored area within the life cycle). Finally in adults (**C**), other genes and other common genes with the precedent phase are utilized (violet color area within the life cycle). In case these animals lose some appendages (or inner organs) during adulthood (**C**), they can regenerate these appendages (yellow color area in (**D**) and within the life cycle). Regeneration takes place by the re-utilization of part or many developing larval-metamorphic genes because they can be functionally integrated with the adult genes. If this integration of adult active and metamorphic genes is not functional, no regeneration or a more limited regeneration occurs.

Using simple models of gene networks in the following discussion I only mean to provide a visual and general indication on the thousands of genes present in different animal genomes [8] capable or not to determine a regenerative process (Figure 3).

In general, the development and anatomical simplicity of the resulting adult phase favors regeneration because of the lower number of “gene regulative networks” necessary to develop simple organisms that have a tissue organization level of complexity in their bodies such as sponges, jellyfish, planarians, ribbonworms. Also in more complex organisms such as seaworms (anellids) or echinoderms or marine chordates, that are master of regeneration, their more complex anatomical organization can be regenerated after injury as they possess ample restructuring processes operating during their metamorphosis, and that can be largely re-used in case of injury [2,4]. The variable regenerative ability present in these animals, from large body regions to minute organs, suggests that these animals re-utilize a number of metamorphic-developmental genes for their partial or broad regeneration when they become adults but are still living in an aquatic environment (Figure 3). Only metamorphic processes that involve large re-modeling of the larval body to reach the sexually mature adult condition determine a high or discrete ability to regenerate as adults. This derives from genomes involved in large restructuring morphogenetic processes that transform a larva into an adult [15,16,17]. In contrast, the simple increase of body size during larval growth after successive molts, such as in numerous crustaceans, insects or some spiders, is not associated with a broad regeneration in adults, aside from body appendages [1,2,3,4,5,6]. This likely derives from the lack of developmental gene networks implicated in broad morphogenetic and restructuring processes in the genomes of these animals operating during their life-cycle. 

**Figure 4:**
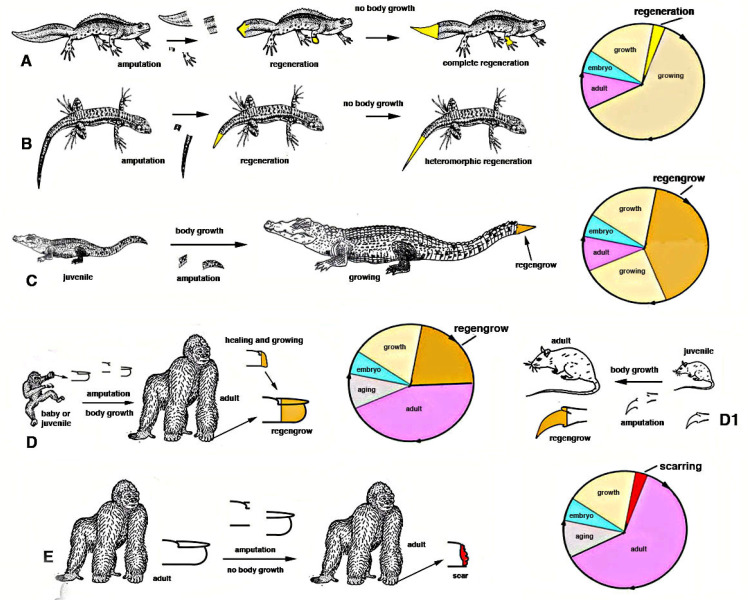
Drawing summarizing the difference between regeneration and *regengrow* in relation to the life cycles (colored circles) of 5 different vertebrates. In (**A**), a newt regenerates the limb and the tail within a short period within the life cycle (yellow wedge within the life cycle), with almost no body growth during this period. In (**B**), a lizard rapidly regenerates (yellow wedge within the circle) its tail so that the somatic growth (growing, pale brown color) is not influential on the process. However the tail is simpler than the original one (heteromorphic regeneration in the yellow section of the life cycle). In (**C**), a young crocodilian loses its tail tip that heals and grows during the somatic growth into an adult crocodilian, a process of *regengrow* (the large orange section of the life cycle). In (**D**) and (**D1**) after the loss of the tip of a digit in an young gorilla, a rhesus monkey, or for a mouse, the digit heals and grows like the rest of the body to become adult. The digital tip is reformed by *regengrow* (the orange section of the life cycle). (**E**), in an adult gorilla or rhesus monkey (like humans or mice), no longer growing, the loss of a digital tips only determines scarring with permanent loss of the digit extremity (the red section of the life cycle, after full gowth).

After injury in some species of juvenile arthropods and vertebrate amniotes, wound healing takes place together with their ongoing somatic growth and the injured or lost organ can apparently regenerate. This occurs for some appendages such as legs, digits or tails [1,11]. However, what derives from true tissue regeneration or from the process of somatic growth of the same tissue is undefined and this mixed healing process is indicated as *regengrow* [18,19,21] (Figure 4).

In *growing* invertebrates or vertebrates various tissues increase their mass due to a more or less continuous production of new cells, from local staminal/reserve cells. For instance a growing squid, a spider or an insect, a fish or a snake, a rat or a child possess muscles that add new (satellite) cells inside the myofiber, contributing to their volumetric increment before reaching the specific full growth of the species. Other processes of addition of new cells also occurs in the new cuticle that is formed from imaginal disks in insects as long as molt occurs, or in growth plates of vertebrate long bones, or in the liver and intestine during somatic growth etc. In case of injury or traumatic loss of muscles, skin, liver etc. in still growing animals, the activation of a wound healing process simply increases the rate of cell proliferation and tissue growth that was already present in these animals. Consequently, what derives from the physiological growth process superimposed to the stimulated healing or broader regeneration becomes undefined, and represents a mixed process indicated as *regengrow*. Regengrow is progressive but requires time to occur, over 4-5% of its life-span [18,19,21], like the growth of that specific animal, and stops when these animals complete their somatic growth. Numerous examples of *regengrow* are reported for vertebrates [18,19], but also numerous terrestrial invertebrates incurs in a similar process. At the end of growth in each species, and during aging in species that manifest this process, the addition of new cells is reduced, halted and only a physiological regeneration (skin, blood, intestine etc.) remains. An injury at this late stage of the life cycle in different species (e.g., after the end of the last molt in a crab or in a grasshopper or after reaching adulthood in a mammal etc.) cannot anymore combine growth (ceased) with wound healing/regeneration, and only scarring can occur with the permanent loss of the injured organ.

In contrast, a true process of regeneration takes a relatively short time (2–3% of the lifetime of a species, at least in vertebrates) such that the process of somatic growth is not influencing or overlapping with the healing (regenerative) process [21]. While *regengrow* takes a long time, even years in some vertebrates to apparently reform the missing organ, regeneration rapidly occurs through proliferation and differentiation of a very large number of new cells that determines the reconstitution of the injured organ. Regeneration can also occur during active somatic growth and also in invertebrate and vertebrate larvae, but requires a short time where the growth contribution is minimum or irrelevant. Regeneration also rapidly occurs in any other phases of the life cycle of regenerative competent species, including adult and even during aging, while *regengrow* is no longer possible at the end of growth in adults [19]. For example, when full somatic growth is reached in an animal, the amputation of a mammalian digit, a crocodilian tail, the leg of a crab or a lobster or a cockroach or a grasshopper or a spider after the last molt, determines scarring or the formation of short outgrowths but not their regeneration (Figure 4).

Chordate regeneration is very likely a basal capability for the phylum like asexual propagation (reproduction), and the fact that extant chordate larvae can broadly regenerate after injury supports this notion [22] (Figure 1). The only vertebrates capable of a variable but generally broad inner organ and appendages regeneration are water-related, fish and amphibians, while amniotes can only heal their wounds or form scars [3,13,15,17]. The only exception among amniotes is the broad regeneration of the tail in numerous lizards, often a short process that takes 2–4% of the entire life-span of a lizard to restore a voluminous organ (1/7th–1/4th of the body mass), but that gives rise to a heteromorphic tail, missing of axial anatomical structures. Therefore, a general conclusion is that after organ loss the more larval-metamorphosis genes recruited in adults of invertebrates or vertebrates, the better the regeneration that takes places (Figure 3).

The above discussion indicates that gene networks present in any genome can be largely or partially re-activated for regeneration in simple or more complex aquatic animals with larval stages and metamorphosis while terrestrial animals, without metamorphosis, cannot re-activate functional gene networks for regenerate their lost organs. The presence of metamorphic events in the life cycle of animals, determined by genes, for organ destruction of larvae and for re-construction of new organs in adults, allows for the re-use of these genes in part for regeneration in adult animals living in water but not on the land [16] (Figure 3). After injury, it is difficult to activate alternative developmental pathways from those utilized during development, especially in complex terrestrial arthropods or vertebrates. Additionally, during leg regeneration in arthropods or tail regeneration in lizards, a process that allows a broad but heteromorphic regeneration, only few developmental genes appear to be re-activated [15,16,17,18,23,24,25]. This suggests that regeneration, like development, requires the function of basic developmental pathways, which have altered during the evolution of different forms of metazoans, resulting in an inability to regenerate [5].

## 3. Regenerative Species Evolved Genomes including Programs for Larvae Development and Metamorphosis

**Figure 5:**
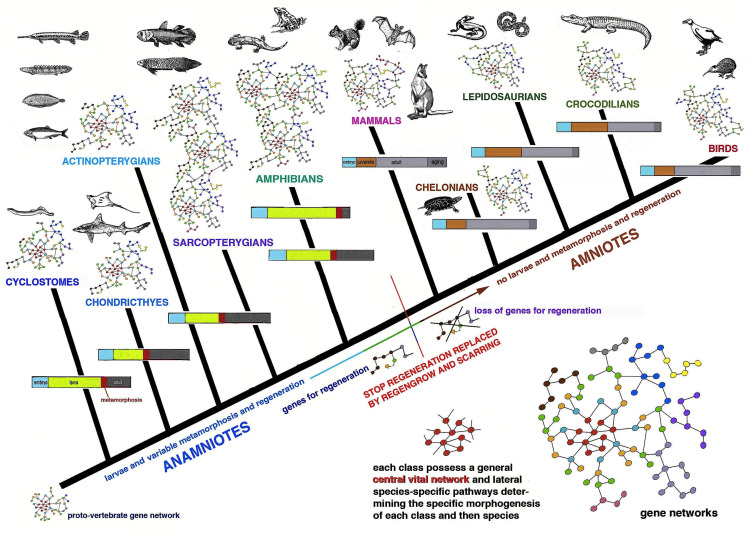
Simple phylogenetic scheme featuring indicative genomes (made by gene networks) for different vertebrates. The progressive phases of the life cycle are here represented by colored bars accounting for the initial embryonic (light blue), larval (green), metamorphosis (dark red) and adult (dark grey) phases for anamniotes (fish and amphibians). For amniotes, the larval and metamorphic phases were lost during evolution for the terrestrial environment, and larvae were replaced by a juvenile and variable growing phase (brown), followed by the adult phase (grey) and, finally, by a short or longer aging phase (dark grey). For each class, a definitive genome, made of a unique gene network, has evolved. Moving from the aquatic environment where the first vertebrates, fish and then amphibians, evolved to the terrestrial environment of amniotes (reptiles, birds and mammals), the loss, deactivation or decoupling of some genes involved in larva development and metamorphosis determined also the loss of regenerative abilities. Gene networks are only idealized as simple mesh of genes or “gene modules” (the dots) in their interactions (the connecting lines).

The genomes of different animals have been acquired during the evolution in each lineage of metazoans and allow their specific phases of development and post-embryonic development during the progression in their specific life cycles (Figure 2 and Figure 5). We should interrogate ourselves and reflect on the biological reasons that motivate regeneration only in some species. Can what we learn from regenerative species really be applied to non-regenerating species? A response should not only look to the genetic and informational molecules but also elaborate on the specific evolutive process (trajectories) that generated different animal species capable or incapable of regeneration (Figure 1 and Figure 5).

**Figure 6:**
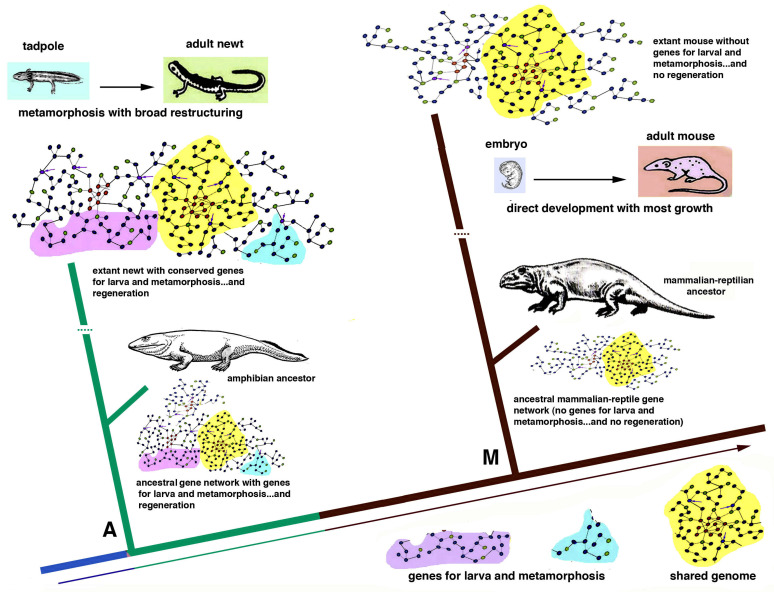
Schematic simple drawing that zooms in between the amphibian and mammalian evolutive lineages in Figure 6 to point out only two examples of amphibian genome evolution (**A**) vs. that of a mammal (**M**). In the newt genome, like its pisciform ancestor, genes for metamorphosis that can be also used for regeneration in case of injury are still present. This is not the case for the mouse where these genes were already missed in its terrestrial reptilian-mammalian ancestor where a direct development (an egg from a blastocyst in the mouse at that time) eliminated the larval stage. Once terminated, an evolutive direction (amphibian or mammalian) formed endpoint genomes, the latter of which cannot change dramatically without determining developmental failure but only incorporate small and viable mutations.

The evolution in each lineage gave rise to species with different genomes but that possess a common region for basic metabolic and genetic functions (basic vital genes, ideally shown in Figure 3C). These are roughly common among most organisms for metabolism, cell division, cell communication and interactions. Other genes work in different networks (schematically indicated as peripheral areas in Figure 3 and Figure 6) for specific functions (development, endocrine, nervous, sensing, movement, reproduction, waste elimination, etc.). Among specific functions I include healing from injuries through a broad or limited regeneration or through scarring.

In the case of vertebrates, broad organ regeneration was lost during the transition from water to land in the Upper Carboniferous, a transition that determined the partial or total loss of the larval stage and of intense metamorphic transformation [14,15,16,17,26]. I hypothesize that some or most of the gene networks that are utilized for larva and metamorphic transformations also functioned for organ regeneration in adults of fish and amphibians, but they were lost or modified for the terrestrial adaptation in amniotes [16] (Figure 5). Even the insertion of only one single, new gene present in amniotes into the genome of a fish can inhibit its ability to regenerate the fin [27], demonstrating that the developmental regenerative program can be easily altered.

**Figure 7:**
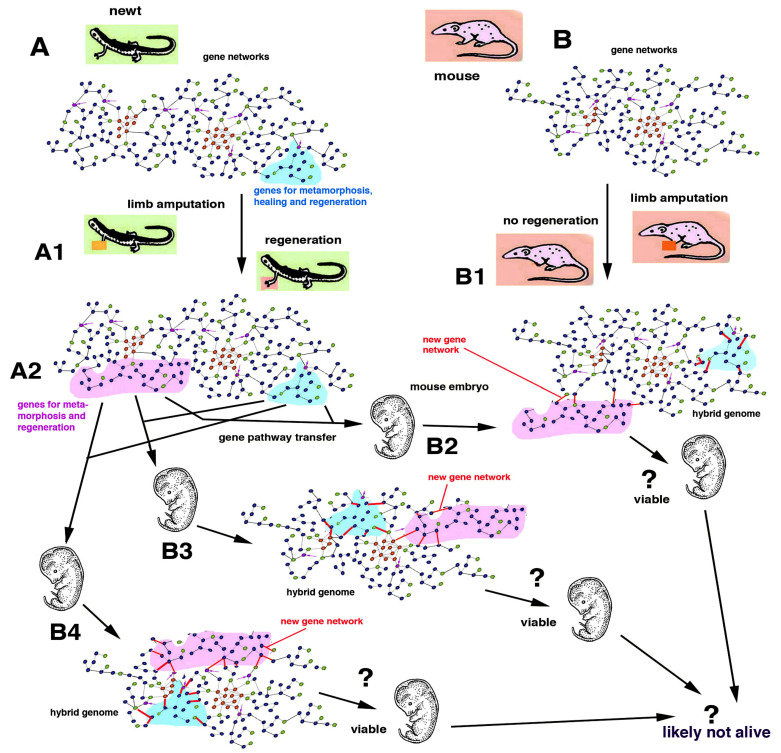
A schematic example of a very hypothetical “regenerative gene therapy” made from the transplant of some genes from a newt (**A**) to a mouse (**B**) in an early embryo. While the newt can regenerate an amputated limb (**A1**), the mouse cannot (**B1**). The insertion of genes utilized, for instance, during limb regeneration in a newt (**A2**) into the complex gene regulative meshwork of a developing mouse (**B2**–**B4**), form a “hybrid genome”. In the example, three different integrations that give rise to new gene networks are shown (the red lines indicated as “new gene network” in (**B2**–**B4**)). These new genomes will likely not be able to integrate into a functional developmental network and the consequent mouse embryos undergo an uncertain fate (?), most likely death or variable degrees of teratomas and cancer. The new gene networks of hybrid genomes cannot be activated as those of the newt since other genes, acquired during mammalian evolution, would interfere with the newt genes to create faulty and likely deadly combinations.

As a theoretical example of gene insertion in different species, we here consider two very different vertebrates, a newt and a mouse, that derived from a common ancestor, about 280–300 million years ago [20] (Figure 6). Like the amphibian ancestor of the Carboniferous Period, the extant newt possesses a genome that includes several gene networks in common with those of a mammalian-reptilian ancestor and in the extant mouse (idealized yellow areas in Figure 6). This “central region of the genome” governs mainly basic viable processes needed for both these species. Other regions of the genome of the newt, but not in the mouse, represented in Figure 6 as located peripherally to the gene network (in pink and light blue colors in Figure 6), act on specific anatomical-physiological characteristics of the newt such as development of a larval stage and metamorphosis. The way morphogenesis occurs in the newt and in the mouse is, however, different in many other gene networks that evolved in amphibians in comparison to others that evolved in mammals. Like its amphibian ancestors, the newt possesses a larval stage with broad metamorphosis that determines a large body-restructuring and a derived broad ability to regenerate appendages and inner organs (Figure 3 and Figure 5). In contrast, the mouse, like its reptilian-mammalian ancestor, has a direct development and the delivered baby mainly grows into a juvenile mouse with no metamorphosis. In case of general injury, an adult mouse can only rapidly heal by scarring [13] or producing new digit tips by *regengrow* if the loss occurred in juveniles (Figure 4D1). The different evolution of the “gene networks” undergone in the two species cannot be reversed by the transplant of genes only involved in regeneration (Figure 6 and Figure 7, see later discussion).

General gene pathways governing regenerative cellular processes in polyps, flatworms, earthworms, starfish and chordates including urodele amphibians will hopefully be deciphered in the future with the goal of applying this knowledge to animals with no regeneration [7,12,24]. This future knowledge will have to face the biological specificity of the developmental processes of non-regenerating animals and the biological limits and perils derived from the introduction of “regenerative gene pathways” within the genomes of embryos or adults in species that have evolved abolishing the gene regulatory mesh-works of regeneration initially present in their ancestors [8] (Figure 5 and Figure 6). The above consideration indicates that the information on the molecular mechanisms of regeneration gained from simple models (hydra and planaria) but also from urodele amphibians and fish, cannot be directly applied to different metazoans, especially to vertebrates, because their evolution followed so many different developmental trajectories that they cannot be modified anymore without introducing unpredictable effects (?) including cancer, teratomas or even monstrosity or, more likely, death of the embryo (Figure 7).

## 4. Problems to Induce Regeneration in Non-Regenerating Animals

Gene therapy functions with genes operating by the terminal phase of a gene pathway, e.g., the origin of an eye pigment (cure for albinism) or of hemoglobin production (cure for thalassemia or sickle cell anemia). In contrast, in the case of developmental pathways and their interactions with other pathways within a gene network, it is difficult to predict the outcome of a gene therapy. To further complicate the entire matter of the genetic material and its manipulations, recent studies have indicated the instability of genomes with the loss of segments of DNA in somatic cells [28,29]. Whether the loss of DNA during evolution may also be connected to the loss of regeneration in some phyla, classes or families of metazoans remains an open question for future investigations. Whatever the case, it is likely that with present and future technologies, the molecular details of the developmental pathways leading to various levels of organ regeneration in urodeles will become known. At that point, some gene therapies might be planned, transferring some key genes or one or more “gene modules” identified in the newt (or axolotl) into (a) the genome of a fertilized egg or the early embryos of a mouse, or (b) into some mouse cells that are implanted into a damaged but not completely lost organ [30,31,32]. In the first case (a), this “early regenerative gene therapy” for curing an organ or limb loss that may occur in adults of mice would be unpredictable since the numerous interactions and consequences on the entire function of a mouse genome would be likely teratogenic and lethal (Figure 7). In fact, gene pathways that have, since at least 280–300 million years ago, evolved to form numerous differences between those of a newt and a mouse cannot be re-assembled anymore. Few experiments have already indicated that to stop regeneration, only the alteration of some (key) genes that lead to change of basic signaling pathways such as Wnt and BMP or other genes is required [2,6,32]. This type of genetic manipulation would very likely produce teratomas and death of the embryo and is consequently not appliable (Figure 7).

In the second case (b), some attempts of gene therapies for tissue regeneration are currently in trial but these therapies insert few specific genes directly into specific injured organs or insert specific genes in cell types that are later transplanted in precise locations of the damaged organs [30,32,33]. The engineered cells proliferate and give rise to a regenerated tissue but do not integrate into the genome of the receiving animal. However, numerous problems must be solved, such as the type of viral or non-viral (e.g., liposomes) vectors and care should be exerted to control that the regenerating tissue does not turn into a tumor. These methods are an extension of those that use stem cells for stimulating tissue regeneration [31,32]. New technologies that utilize innovative genetic manipulations such as “tissue regeneration enhancer elements” and are delivered to localized cells of specific organs are currently under active study [33].

Some improvements in healing can be obtained in non-regenerating animals, including humans, using these methods when the technology will be perfected. Regenerative gene therapies transferring genes such as *Wnt*, *BMP*, *VEGF*, *PDGF*, *IGF*, *TGGb1* etc. are in trial for bone, teeth and cartilage regeneration, as are *microdystrophin* and *Cyclin A2* genes for muscle regeneration, *CDGF*, *BDGF*, *GDNF* genes for neural regeneration, *KGF*, *IGF1* genes for skin regeneration and so forth [32]. For inducing digit and limb regeneration in mice, other genes transfer (*Lin28*) has been attempted [31]. Whether this gene therapy will last in the injected cells in order to be able to induce the regeneration of large organs such as limbs, heart, intestine, kidneys, etc. remains uncertain. This results from the lack of information on the fate of the engineered cells among adult tissues and under immunological check-up from the receiving body. Furthermore, the formation of a possible blastema should keep the tissue wet since regeneration only occurs in a liquid environment, as previously indicated [21]. Other technological avenues (c) should be taken for alleviating inner organ, leg and arm loss based on tissue engineering or combining bio-engineered prostheses [31,34] and not acting on invariant animal genomes derived from long evolutive differences (Figure 7). The functionality of organs after injury or pathological damage could be replaced efficiently only through improvements in the technology of prostheses and organ implants which are not impinging on genetic problems but mainly have immunological problems related to the acceptance of these organs.

## 5. Conclusions

The present MS has briefly presented a new hypothesis that tries to explain the distribution of regeneration among animals. This depends on their biological complexity, genomes, environment where their genes can be expressed and their life cycles. The hypothesis concludes that only animals with large metamorphic transitions during the life cycle can broadly but variably regenerate when they are adults. The present analysis indicates that, due to the biological limits derived from the different evolutionary directions taken in different phyla of metazoans to adapt to an aquatic, semiaquatic or terrestrial environment, the induction of regeneration in non-regenerative terrestrial animals would be extremely difficult and with uncertain results. This conclusion must, however, wait for future clarifications on developmental gene regulatory pathways present in some phyla of metazoans. Only by mastering this information will next generations of biologists and physicians be able to insert and manipulate regenerative mechanisms in non-regenerating animals in order to select which gene insertions could still give rise to viable organs [33]. The future of regenerative medicine must consider basic biological principles before using genes active in regenerating animals for non-regenerating animals.

## Data Availability

Not applicable.
